# Enabling precision medicine with biomarkers of response to treatment in atopic dermatitis: where are we now? A narrative review

**DOI:** 10.3389/fmed.2025.1574697

**Published:** 2025-04-10

**Authors:** Andrea Chiricozzi, Anna Levi, Chiara Palladino, Giampiero Girolomoni

**Affiliations:** ^1^Unit of Dermatology, Department of Medical and Surgical Sciences, IRCCS A. Gemelli University Polyclinic Foundation, Rome, Italy; ^2^Section of Dermatology, Department of Medicine and Translational Surgery, Sacred Heart Catholic University, Rome, Italy; ^3^AbbVie Srl, Rome, Italy; ^4^Department of Medicine, Section of Dermatology, University of Verona, Verona, Italy

**Keywords:** atopic dermatitis, targeted therapies, predictive biomarkers, precision medicine, personalized approach

## Abstract

Atopic dermatitis (AD) is a complex systemic disease characterized by high heterogeneity both at clinical and pathophysiology levels. While advances in drug development toward a more targeted approach are made, treatment and management of AD patients are still happening according to the one-size-fits-all approach. To enhance precision medicine in AD and improve care, identifying predicting factors of response to therapy driving tailored treatments will be of utmost importance. Here, we discuss the available evidence regarding predictive biomarkers, as well as their possible and still debated impact in clinical practice.

## Introduction

Atopic dermatitis (AD) is a complex and heterogeneous systemic disease (1, 2) and the most common chronic skin disorder (3), that affects a large number of people at any age (4). The heterogeneity of AD lies both at pathophysiology and clinical levels (5), leading to a classification into different phenotypes or endotypes (6).

Clinical and disease characteristics of AD allow for a stratification into different disease phenotypes based on age, age of onset, ethnicity, and presence of other atopic diseases, such as allergic rhinitis and asthma (5, 7, 8). However, a more recent categorization, based on the underlying molecular mechanisms known as endotypes, has been proposed (9).

The high complexity and heterogeneity of these molecular mechanisms in AD, also determining the different clinical phenotypes, might explain the different responses to treatments among patients (5, 10). Although endotypes could guarantee a more efficient classification of AD, the differences in the immune profile across patient groups have not been fully elucidated yet (9). Moreover, the definition of the immune profile (or patients’ endotypes) and the identification of a panel of multiple biomarkers (11) may allow patient clustering into subgroups, and thus the tailoring of the therapeutic approach through the use of more targeted therapeutic approaches (5).

The recent introduction of targeted biologic and systemic Janus kinase (JAK) inhibitors has profoundly changed the therapeutic landscape in AD (12), offering the possibility of more personalized approaches based on patient-specific biomarkers, which are defined as measurable indicators of some biological state or condition evaluated on different biological materials, including whole blood, plasma, serum, and tissue samples, on which molecular, genetic, or morphologic data can be analyzed (10).

Biomarkers, when assessed early, may predict therapeutic responses to drugs and, therefore, identify patient subsets that would benefit most from a treatment (10, 11). However, nowadays, AD is still a clinical diagnosis based on physicians’ clinical experience and experts agree on the unmet need to identify a list of reliable biomarkers useful for patients’ stratification and treatment monitoring (13).

The state-of-the art knowledge about biomarkers in AD was recently reviewed by Renert-Yuval et al. (10), Bakker et al. (11), and Park et al. (14). Here, we discuss the most up-to-date knowledge about predictive biomarkers. For this purpose, we selected the most clinically impactful papers (including original articles, narrative, and systematic reviews, expert consensus, and expert opinions) published on this topic in journals indexed by PubMed, between 2016 and 2024.

## Biomarkers by treatment options

Although clear suggestions for clinical practice are still lacking, several biomarkers aiming at predicting a therapeutic response in patients with AD have been studied.

### CCL22

Glickman and colleagues compared clinical and molecular outcomes of different treatments for AD and showed that baseline CCL22 expression was correlated with clinical improvement across multiple studies at various time points without being drug-specific (15).

### CXCL2

The baseline levels of Th17 cell-related cytokine CXCL2 predicted response to therapy at week 16 in patients treated with dupilumab which targets the IL-4 receptor alpha chain that inhibits both IL-4 and IL-13-related cascades (15). However, a clear molecular explanation of this association is lacking and no further studies have corroborated the role of CXCL2 as biomarker yet.

### IgE

Based on the IgE levels and IgE specific to protein antigens, AD was classified into extrinsic and intrinsic subtypes: the first characterized by high IgE and type 2 cytokines levels, and the second by normal IgE and lower type 2 cytokines levels (9). Measurement of IgE levels has been a routine practice in diagnosing AD for a long time, possibly because IgE levels could be high in the extrinsic AD form and clinicians often consider the serum total IgE levels as a biomarker of AD severity (10). Despite the historical association between high IgE levels and type 2 cytokines pathway, patients’ responses to dupilumab were not clearly correlated to baseline IgE levels (10), while changes in the IgE levels during the course of treatment might be a tool to monitor therapeutic efficacy according to other evidence (16). Also, no significant changes in total and specific IgE levels were observed after 16 weeks of upadacitinib treatment during a phase 2 study, while still proving its efficacy at clinical level (17). Hence, IgE levels do not appear to be a candidate for predictive biomarkers.

### Dipeptidyl peptidase-4 (DPP-4) and periostin

Baseline high levels of serum of DPP-4 and periostin, indicators of increased activity of IL-13, have been identified as potential biomarkers of a good response to tralokinumab, a fully human mAb blocking IL-13 (18). In an exploratory analysis of a phase 2b study, Wollenberg and colleagues observed that in patients with moderate-to-severe AD and a baseline higher concentration of PPD-4 and periostin, tralokinumab led to greater changes from the baseline on the Eczema Area Severity Index (EASI) (18).

### IL5RA, CCL23, and eosinophil count

Upadacitinib, an oral Janus kinase (JAK)-1 inhibitor significantly reduced, among other mediators, the levels of serum IL5RA and CCL23 as early as week 1 of treatment (19). A significant change was also a reduction in absolute eosinophils count after week 2 of upadacitinib treatment, maintained through week 24 (19). Hagino et al. showed that total eosinophil count correlates with EASI and PP-NRS during upadacitinib treatment over 48 weeks (20), indicating its potential as a biomarker reflecting treatment responses. IL5RA and CCL23 genes were upregulated in the eosinophil-high endotype (21). It could be interesting to assess whether patients with a high-eosinophil profile might benefit the most from treatment with upadacitinib. In addition, a real-world study from Zheng et al. showed significant eosinophil count reduction after 4 weeks of abrocitinib treatment in 47 patients (22).

### IL-22

According to Brunner et al. baseline IL-22 expression can be a biomarker able to predict a therapeutic response to fezakinumab. Results from a phase 2a clinical trial showed that among patients stratified based on baseline skin IL-22 mRNA expression, those with high IL-22 baseline levels were more likely to improve both at clinical and transcriptomic level. Fezakinumab did not meet the primary endpoint of the study at week 12 (23). However, also JAKi targets IL-22: IL-22-attracting chemokines were significantly reduced with upadacitinib treatment as early as week 2, suggesting that upadacitinib may have early and robust effects on Th22 axes (17). Also abrocitinib reduced Th22-associated genes (S100A8/9/12) in a dose-dependent manner, starting from week 2 throughout week 12 (24).

## The role of AD pathogenetic pathways in predicting response

Four differentiated clusters of patients have been identified by using a data-driven approach based on serum biomarkers (6). Each cluster was characterized by a specific serum biomarker profile, implying that a distinct underlying immunopathologic pathway drives each cluster. Two of these clusters were characterized by a Th2-dominated biomarker profile (i.e., “Th1/Th2/Th17-dominant” and “Th2/Th22/PARC-dominant”), with high levels of Th2-related cytokines, suggesting that patients in these clusters would be ideal candidates for Th2-blockers (6). An exploratory analysis of the phase 2b study with tralokinumab found a better treatment response in patients with high levels of DPP-4 and periostin (18). Pediatric AD is characterized by a Th2, Th9, and Th17 higher polarization, thereby suggesting that the current biologic drugs targeting Th2 cytokines would be more effective in children than in adults, who, instead, have a more pronounced Th22 dominant T-cell response (5). Similarly, the Th17 higher contribution reported in the Asian population would support an effect of anti-IL-17 therapy, such as secukinumab, in order to treat AD in this ethnic group (5, 25). However, a phase 2b randomized double-blind controlled study failed to show a clinical benefit of secukinumab in adult AD, and several cases of anti-IL-17-induced AD have been reported (25). Therefore, the IL-17 pathway, although may contribute, does not appear to be essential to AD pathogenesis. Indeed, it is well known that together with Th2 and Th22 inflammatory pathways, the involvement of Th1 and Th17 play also a role in AD and varies by patient race and age (24). Upadacitinib, beyond the clinical efficacy, downregulated key AD genes associated of multiple pathways such as Th2 (CCL17), Th1 (CXCL10), and Th22 (S100A9/12, PI3) potentially supporting a more complete disease control (17). Abrocitinib showed similar results, downregulating genes associated with inflammation, epidermal hyperplasia, Th2 and Th22 immune responses in the skin of patients with moderate-to-severe AD (24).

## Sources of biomarkers

Biomarkers differ according to the source of biological material: (i) genomic information, (ii) transcriptomic profiles, (iii) proteins from body fluids or tape stripping, and (iv) morphological information (10), with possible sources of biomarkers being blood, tape strips of skin, and skin biopsy (26). Skin biopsy, although invasive, accurately reflects disease severity and often detects AD biomarkers not measurable by blood tests. The tape strip sampling technique, a non-invasive approach that collects stratum corneum proteins, has shown promising results (10, 27, 28). Not all biomarkers discussed here can be measured in both serum and skin (10). A minimally invasive approach guarantees repeated assessments of biomarkers and would be ideal to define the therapeutic outcome and improve patients’ adherence.

## Clinical predictive factors

Given the current landscape of AD, reliable biomarkers are necessary; hence, if blood-based or skin-based molecular markers will not bring these tools to clinical practice, then physicians could leverage on clinical predictive factors, based on patients’ signs, symptoms, and comorbidities (29). A recent *post hoc* analysis showed that baseline body surface area affecting 10–40% and severe itch (i.e., Numerical Rating Scale ≥7) were the strongest predictors of the response to the oral JAK 1/2 inhibitor baricitinib, in adults with moderate-to-severe AD (30). Likewise, a retrospective study on patients with moderate-to-severe AD, showed a greater likelihood of response to upadacitinib 15 mg in those with lower EASI (<24) and older age and to upadacitinib 30 mg for individuals with lower levels of IgE and LDH (31). AD localization in exposed areas at the baseline and AD persistency in the head/neck, a known phenomenon in difficult-to-treat AD patients, may also drive treatment choice (32). Furthermore, *de novo* appearance of dupilumab-associated head and neck dermatitis, now recognized as a distinct entity, can be successfully resolved with upadacitinib (33) and seems to be associated with normalization of IL-4/IL-13 downstream activity markers such as CCL13, CCL17, CCL18, and CCL26, but also with a strong increase in type 22-associated inflammation, enhanced keratinocyte activation, and IL-22 receptor upregulation (34).

## The relevance of predictive biomarkers in daily practice

The tailorization of the therapeutic approach and predictable response of each drug (or class of agents) for a certain patient subtype, would be impactful because it could help lowering the economic burden of targeted therapies using those that likely could be more successful for that specific endotype. The identification of patients potentially responding to one selective agent could increase treatment compliance avoiding switching a time prior to achieve a valid and satisfactory response.

## Conclusions and future outlook

The present therapeutic scenario for AD, including the newly developed biologic and targeted agents, highlights the importance of overcoming the “one-size-fits-all” approach toward a precision medicine one, especially for such a multifactorial and complex disease like AD. There is a need for the identification and validation of reliable biomarkers to predict and monitor the treatment response as objective tools in daily clinical practice: Real-world studies in which single biomarkers or biomarkers profiles (i.e., determined by clusters of biomarkers) are measured in relationship with clinical scoring systems are lacking. Here, we have described few example of candidate biomarkers that might predict a response to treatments. However, no one is currently applied in routine clinical settings. A possible limitation to this hard search for valid biomarkers could be found in the lack of stratification of the studied populations into the responder/non-responder.

Our work wants to draw the attention of the scientific community on this theme, ultimately aiming at improving AD management and advancing the standard of care. Further research will be pivotal for clarifying whether molecular biomarkers of therapeutic response will ever represent a practical tool assisting dermatologist in the selection of the best treatments in patients with AD.

## References

[1] Oliveira C Torres T. More than skin deep: the systemic nature of atopic dermatitis. Eur J Dermatol. (2019) 29:250–8. doi: 10.1684/ejd.2019.3557, PMID: 31122909

[2] Thijs JL Strickland I Bruijnzeel-Koomen CAFM Nierkens S Giovannone B Knol EF. Serum biomarker profiles suggest that atopic dermatitis is a systemic disease. J Allergy Clin Immunol. (2018) 141:1523–6. doi: 10.1016/j.jaci.2017.12.991 29410314

[3] Weidinger S Beck LA Bieber T Kabashima K Irvine AD. Atopic dermatitis. Nat Rev Dis Primers. (2018) 4:1. doi: 10.1038/s41572-018-0001-z, PMID: 29930242

[4] Maurelli M Chiricozzi A Peris K Gisondi P Girolomoni G. Atopic dermatitis in the elderly population. Acta Derm Venereol. (2023) 103:adv13363. doi: 10.2340/actadv.v103.13363, PMID: 38095061 PMC10753596

[5] Bieber T D'Erme AM Akdis CA Traidl-Hoffmann C Lauener R Schappi G. Clinical phenotypes and endophenotypes of atopic dermatitis: where are we, and where should we go? J Allergy Clin Immunol. (2017) 139:S58–64. doi: 10.1016/j.jaci.2017.01.008, PMID: 28390478

[6] Bakker DS Nierkens S Knol EF Giovannone B Delemarre EM van der Schaft J. Confirmation of multiple endotypes in atopic dermatitis based on serum biomarkers. J Allergy Clin Immunol. (2021) 147:189–98. doi: 10.1016/j.jaci.2020.04.062, PMID: 32526312

[7] Werfel T Allam JP Biedermann T Eyerich K Gilles S Guttman-Yassky E. Cellular and molecular immunologic mechanisms in patients with atopic dermatitis. J Allergy Clin Immunol. (2016) 138:336–49. doi: 10.1016/j.jaci.2016.06.010, PMID: 27497276

[8] Girolomoni G de Bruin-Weller M Aoki V Kabashima K Deleuran M Puig L. Nomenclature and clinical phenotypes of atopic dermatitis. Ther Adv Chronic Dis. (2021) 12:20406223211002979. doi: 10.1177/20406223211002979 33854747 PMC8010850

[9] Tokura Y Hayano S. Subtypes of atopic dermatitis: from phenotype to endotype. Allergol Int. (2022) 71:14–24. doi: 10.1016/j.alit.2021.07.003, PMID: 34344611

[10] Renert-Yuval Y Thyssen JP Bissonnette R Bieber T Kabashima K Hijnen D. Biomarkers in atopic dermatitis-a review on behalf of the international eczema council. J Allergy Clin Immunol. (2021) 147:1174–1190.e1. doi: 10.1016/j.jaci.2021.01.013, PMID: 33516871 PMC11304440

[11] Bakker D de Bruin-Weller M Drylewicz J van Wijk F Thijs J. Biomarkers in atopic dermatitis. J Allergy Clin Immunol. (2023) 151:1163–8. doi: 10.1016/j.jaci.2023.01.019, PMID: 36792449

[12] Wollenberg A Christen-Zach S Taieb A Paul C Thyssen JP de Bruin-Weller M. ETFAD/EADV eczema task force 2020 position paper on diagnosis and treatment of atopic dermatitis in adults and children. J Eur Acad Dermatol Venereol. (2020) 34:2717–44. doi: 10.1111/jdv.16892, PMID: 33205485

[13] Patrizi A Costanzo A Patruno C Busa VM Chiricozzi A Girolomoni G. Unmet needs in atopic dermatitis management: an expert consensus. J Dermatolog Treat. (2022) 33:2459–65. doi: 10.1080/09546634.2021.1967267, PMID: 34445932

[14] Park CO Kim SM Lee KH Bieber T. Biomarkers for phenotype-endotype relationship in atopic dermatitis: a critical review. EBioMedicine. (2024) 103:105121. doi: 10.1016/j.ebiom.2024.105121, PMID: 38614010 PMC11021839

[15] Glickman JW Han J Garcet S Krueger JG Pavel AB Guttman-Yassky E. Improving evaluation of drugs in atopic dermatitis by combining clinical and molecular measures. J Allergy Clin Immunol Pract. (2020) 8:3622–3625.e19. doi: 10.1016/j.jaip.2020.07.015, PMID: 32702518

[16] Huang TH Chen YC Lin SY Chiu SH Yang TT Chiu LW. Treatment of atopic dermatitis with dupilumab in Taiwan: dynamic changes of IgE levels as a potential response biomarker. Eur J Dermatol. (2019) 29:658–9. doi: 10.1684/ejd.2019.3661 31903962

[17] Guttman-Yassky E Pavel AB Silverberg J Weidinger S Parmentier J Teixeira HD. Eosinophil count, serum CCL17/18/26 and immunoglobulin E levels in atopic dermatitis: upadacitinib phase 2 study analysis. World Allergy Organ J. (2020) 13:100429. doi: 10.1016/j.waojou.2020.100429

[18] Wollenberg A Howell MD Guttman-Yassky E Silverberg JI Kell C Ranade K. Treatment of atopic dermatitis with tralokinumab, an anti-IL-13 mAb. J Allergy Clin Immunol. (2019) 143:135–41. doi: 10.1016/j.jaci.2018.05.029, PMID: 29906525

[19] Guttman-Yassky E. Gudjonsson J. E. Kabashima K. Bi Y. Teixeira H. Parmentier J.. (2022). Effect of Upadacitinib on cutaneous transcriptomic and systemic proteomic dysregulation in patients with moderate-to-severe atopic dermatitis. 31^st^ Congress of the European Academy of Dermatology and Venerology.

[20] Hagino T Hamada R Yoshida M Fujimoto E Saeki H Kanda N. Total eosinophil count as a biomarker for therapeutic effects of upadacitinib in atopic dermatitis over 48 weeks. Front Immunol. (2024) 15:1365544. doi: 10.3389/fimmu.2024.1365544, PMID: 38745653 PMC11091278

[21] Mobus L Rodriguez E Harder I Boraczynski N Szymczak S Hubenthal M. Blood transcriptome profiling identifies 2 candidate endotypes of atopic dermatitis. J Allergy Clin Immunol. (2022) 150:385–95. doi: 10.1016/j.jaci.2022.02.001, PMID: 35182548

[22] Li Z Wang Y Wu Y Yin H Wang S Wu H. Real-world efficacy and safety of Abrocitinib in Chinese atopic dermatitis patients: a single-center prospective study. Allergy. (2025). doi: 10.1111/all.16495, PMID: 39927851

[23] Brunner PM Pavel AB Khattri S Leonard A Malik K Rose S. Baseline IL-22 expression in patients with atopic dermatitis stratifies tissue responses to fezakinumab. J Allergy Clin Immunol. (2019) 143:142–54. doi: 10.1016/j.jaci.2018.07.028, PMID: 30121291

[24] Guttman-Yassky E Facheris P Gomez-Arias PJ Del Duca E Da Rosa JC Weidinger S. Effect of abrocitinib on skin biomarkers in patients with moderate-to-severe atopic dermatitis. Allergy. (2023) 79:1258–70. doi: 10.1111/all.15969, PMID: 38108208

[25] Ungar B Pavel AB Li R Kimmel G Nia J Hashim P. Phase 2 randomized, double-blind study of IL-17 targeting with secukinumab in atopic dermatitis. J Allergy Clin Immunol. (2021) 147:394–7. doi: 10.1016/j.jaci.2020.04.055, PMID: 32428528

[26] Yu L Li L. Potential biomarkers of atopic dermatitis. Front Med. (2022) 9:1028694. doi: 10.3389/fmed.2022.1028694 PMC9712451 36465933

[27] Koppes SA Brans R Ljubojevic Hadzavdic S Frings-Dresen MH Rustemeyer T Kezic S. Stratum Corneum tape stripping: monitoring of inflammatory mediators in atopic dermatitis patients using topical therapy. Int Arch Allergy Immunol. (2016) 170:187–93. doi: 10.1159/000448400, PMID: 27584583 PMC5296885

[28] He H Olesen CM Pavel AB Clausen ML Wu J Estrada Y. Tape-strip proteomic profiling of atopic dermatitis on Dupilumab identifies minimally invasive biomarkers. Front Immunol. (2020) 11:1768. doi: 10.3389/fimmu.2020.01768, PMID: 32849633 PMC7423990

[29] Flohr C. How we treat atopic dermatitis now and how that will change over the next 5 years. Br J Dermatol. (2023) 188:718–25. doi: 10.1093/bjd/ljac116, PMID: 36715500

[30] Thyssen JP de Bruin-Weller M Costanzo A Grond S Schuster C Liu C. Baseline body surface area and itch severity define response to Baricitinib in patients with moderate-to-severe atopic dermatitis at week 16. Adv Ther. (2023) 40:3574–87. doi: 10.1007/s12325-023-02528-8, PMID: 37332021 PMC10329959

[31] Hagino T Yoshida M Hamada R Saeki H Fujimoto E Kanda N. Predictive factors for responders to upadacitinib treatment in patients with atopic dermatitis. J Dermatolog Treat. (2024) 35:2310643. doi: 10.1080/09546634.2024.2310643, PMID: 38297496

[32] Vittrup I Krogh NS Larsen HHP Elberling J Skov L Ibler KS. A nationwide 104 weeks real-world study of dupilumab in adults with atopic dermatitis: ineffectiveness in head-and-neck dermatitis. J Eur Acad Dermatol Venereol. (2023) 37:1046–55. doi: 10.1111/jdv.18849, PMID: 36606551

[33] Kozera E Flora A Stewart T Gill K Xu J De La Vega MA. Dupilumab-associated head and neck dermatitis resolves temporarily with itraconazole therapy and rapidly with transition to upadacitinib, with Malassezia-specific immunoglobulin E levels mirroring clinical response. J Am Acad Dermatol. (2023) 88:255–7. doi: 10.1016/j.jaad.2022.05.021, PMID: 35588929

[34] Bangert C Alkon N Chennareddy S Arnoldner T Levine JP Pilz M. Dupilumab-associated head and neck dermatitis shows a pronounced type 22 immune signature mediated by oligoclonally expanded T cells. Nat Commun. (2024) 15:2839. doi: 10.1038/s41467-024-46540-0, PMID: 38565563 PMC10987549

